# Mitochondrial Dysfunction in Peripheral Blood Mononuclear Cells as Novel Diagnostic Tools for Non-Alcoholic Fatty Liver Disease: Visualizing Relationships with Known and Potential Disease Biomarkers

**DOI:** 10.3390/diagnostics13142363

**Published:** 2023-07-13

**Authors:** Emirena Garrafa, Agnese Segala, Marika Vezzoli, Emanuela Bottani, Barbara Zanini, Alice Vetturi, Renata Bracale, Chiara Ricci, Alessandra Valerio

**Affiliations:** 1 Department of Laboratory Diagnostics, ASST Spedali Civili, 25123 Brescia, Italy; 2 Department of Molecular and Translational Medicine, University of Brescia, 25123 Brescia, Italy; agnese.segala@unibs.it (A.S.); marika.vezzoli@unibs.it (M.V.); emanuela.bottani@univr.it (E.B.); a.vetturi001@studenti.unibs.it (A.V.); 3 Department of Clinical and Experimental Sciences, University of Brescia, 25123 Brescia, Italy; barbara.zanini@unibs.it (B.Z.); chiara.ricci@unibs.it (C.R.); 4 Department of Medicine and Sciences for Health, Molise University, 86100 Campobasso, Italy; bracale@unimol.it; 5 Division of Gastroenterology, ASST Spedali Civili, 25123 Brescia, Italy

**Keywords:** non-alcoholic fatty liver disease, mitochondrial bioenergetics, peripheral blood mononuclear cells, correlation plot, random forest, relative variable importance

## Abstract

Non-alcoholic fatty liver disease (NAFLD) is a health emergency worldwide due to its high prevalence and the lack of specific therapies. Noninvasive biomarkers supporting NAFLD diagnosis are urgently needed. Liver mitochondrial dysfunction is a central NAFLD pathomechanism that changes throughout disease progression. Blood-cell bioenergetics reflecting mitochondrial organ dysfunction is emerging for its potential applications in diagnostics. We measured real-time mitochondrial respirometry in peripheral blood mononuclear cells (PBMCs), anthropometric parameters, routine blood analytes, and circulating cytokines from a cohort of NAFLD patients (N = 19) and non-NAFLD control subjects (N = 18). PBMC basal respiration, ATP-linked respiration, maximal respiration, and spare respiratory capacity were significantly reduced in NAFLD compared to non-NAFLD cases. Correlation plots were applied to visualize relationships between known or potential NAFLD-related biomarkers, while non-parametric methods were applied to identify which biomarkers are NAFLD predictors. Basal and ATP-linked mitochondrial respiration were negatively correlated with triglycerides and fasting insulin levels and HOMA index. Maximal and spare respiratory capacity were negatively correlated with IL-6 levels. All the mitochondrial respiratory parameters were positively correlated with HDL-cholesterol level and negatively correlated with fatty liver index. We propose including blood cell respirometry in panels of NAFLD diagnostic biomarkers to monitor disease progression and the response to current and novel therapies, including mitochondrial-targeted ones.

## 1. Introduction

Non-alcoholic fatty liver disease (NAFLD), characterized by excessive liver fat accumulation, has become the most common chronic liver disorder globally. It has been estimated that excessive accumulation of hepatocyte lipids in the absence of significant alcohol consumption occurs in approximately 25% of adults [1]. NAFLD encompasses a disease continuum evolving from liver steatosis, with or without mild inflammation, to non-alcoholic steatohepatitis (NASH), with necroinflammation and fibrosis [1]. Due to its high prevalence, NAFLD is emerging as a major cause of end-stage liver disease, primary liver cancer, and liver transplantation—with substantial health and economic burden—and is the most rapidly increasing cause of liver-related mortality [1].

The standard for NAFLD diagnosis is a clinicopathological correlation requiring the absence of known causes for secondary hepatic fat accumulation, such as significant alcohol consumption, use of steatogenic medication, or hereditary disorders, together with the histological confirmation of steatosis in more than 5% of hepatocytes by liver biopsy [2].

Percutaneous liver biopsy is an invasive procedure with severe and potentially life-threatening complications, including hemorrhage, infection, injury to adjacent viscera, and pneumothorax [3]. The biopsy is prone to significant sampling bias since only a tiny portion of the total mass of the liver is obtained from the needle liver biopsy. Notably, the liver is not affected uniformly (especially regarding features such as fibrosis), so the sample cannot be assumed to represent the entire liver. A significant limitation is that the histological analysis is influenced by the skill and experience of the reading pathologist and is subject to intra- and inter-observer variability [3]. Therefore, this invasive procedure is unsuitable for widespread use to assess disease stage and progression or therapeutic response [1]. In a provoking way, liver biopsy has been termed a “snapshot” taken at a single moment of a long-lasting chronic disease [4], highlighting the need for noninvasive techniques to diagnose the disease at different stages.

Noninvasive imaging modalities for the detection of liver steatosis, such as ultrasonography (US), computed tomography (CT), and magnetic resonance imaging (MRI), have been implemented [2]. Though presenting sensitivity limitations, variability depending on the patient’s characteristics (e.g., obesity), and operator dependency, abdominal US is the most applied imaging technique in routine practice. It is cheaper and more widely available than the NAFLD imaging gold standard (MRI), which requires waiting time and specialized staff to perform and evaluate the results [1,2].

Many authors have also investigated the role of biochemical markers to identify NAFLD patients and their progression, which could help in triaging patients to discriminate against those who should undergo rapid imaging or biopsy to allow prompt initiation of treatment. A mild to moderate elevation of serum aminotransferases with the AST/ALT (aspartate aminotransferase/alanine aminotransferase) ratio usually less than one in the absence of advanced fibrosis [5] has been considered typical of NAFLD. The utility of this observation in the current practice is limited because (i) increased transaminases are not highly specific for steatosis and can be found in other clinical situations; (ii) transaminases are frequently found within a normal range in NAFLD patients; (iii) their levels can fluctuate, and do not necessarily correlate with liver histology. A more recent study has combined the threshold of ALT levels (19 IU/L) with triglyceride levels (101 mg/dL) to be used for the screening of NAFLD [6], without overcoming the limitations mentioned above.

Recent efforts have been reported in the search for new potential serum biomarkers as noninvasive selective indicators of NAFLD. Inflammatory biomarkers have been found to be associated with NAFLD [7]. In particular, circulating cytokines, like tumor necrosis factor-α (TNF-α) and interleukin (IL)-6 (IL-6), were observed to correlate with the severity of inflammation and fibrosis [8]. However, limited data are available on these markers’ accuracy and clinical usefulness for noninvasive diagnosis, and further research is ongoing [8].

In a multifactorial disease such as NAFLD, it seems unlikely that observed pathologies could be attributed to changes in the expression or release of a single molecule. More likely, it results from the concerted actions of large numbers of molecules over a prolonged period that could be detected, in part, by using biomarker panels. Various score systems are recognized by European guidelines [2], including the widely used fatty liver index [9], the FibroTest/Fibrosure ratio [10], the NAFLD fibrosis score [5], and others. However, these methods are more helpful in guiding disease management in patients with an advanced disease rather than in primary care settings, as their sensitivity and specificity are lower in the earlier stages of the disease [11]. Thus, despite the efforts, none of the methods mentioned above are completely acceptable for the early diagnosis and follow-up of NAFLD patients, and novel approaches are required.

Mitochondrial dysfunction is considered a notable contributor to the development of NAFLD [12,13]. Free fatty acids (FFAs) and triglyceride accumulation in the liver is associated with increased β-oxidation in hepatocytes, eventually leading to electron transport chain overload and disruption, with consequent ROS overproduction and mitochondrial damage. The increased production of proinflammatory cytokines, including IL-6 [14], further aggravates this vicious cycle [13]. Some conflicting evidence exists in the literature about the timing and role of mitochondrial derangements in NAFLD pathophysiology. Koliaki and colleagues showed an increased oxidative capacity in hepatic mitochondria isolated from patients at early NAFLD stages and significant downregulation in NASH patients [15]. A possible interpretation is that when simple steatosis proceeds to NASH, the metabolic flexibility of hepatic mitochondria, including oxidative capacity and redox defenses, is progressively lost [13]. Although much remains to be explored in this controversial topic, liver mitochondria become ineffective in NAFLD patients, with reduced respiratory activity and ATP formation.

Circulating blood cells can be obtained by minimally invasive methodology. Recent studies are investigating their potential as biomarkers of systemic bioenergetic function in human subjects [16]. Peripheral blood mononuclear cells (PBMCs), a heterogeneous cell population comprised of monocytes, T and B lymphocytes, dendritic cells, and natural killer cells, provide an easily accessible source of viable mitochondria and are the most frequently used to this aim [16]. The advent of extracellular flux analysis techniques, allowing dynamic measures of mitochondrial function by the sequential addition of defined metabolic inhibitors, has made it possible to demonstrate altered PBMC bioenergetics in several chronic diseases [16,17]. With the aim of making our contribution to the search for comprehensive and noninvasive NAFLD diagnostic methods, we investigated mitochondrial respirometry parameters in PBMCs from NAFLD patients and non-NAFLD control subjects and analyzed their relationships with established or potential NAFLD-related biomarkers.

## 2. Materials and Methods

### 2.1. Study Design and Participants

Voluntary controls and patients were recruited in the context of a larger non-randomized clinical trial, approved by the Ethical Committee of Brescia District (#NP2587) and registered on clinicaltrial.gov as “Non-Alcoholic Fatty Liver Disease: Nutritional Epidemiology and Lifestyle Medicine” with the identifier number NCT03300661. Selection criteria were the absence of a severe clinical condition, pregnancy or breastfeeding, and absence of liver disease and related risk factors (alcohol heavy intake, hepatitis virus infection, hemochromatosis, and others). The sub-study described in the present manuscript included a cohort of 37 subjects aged 40–59 years: 19 NAFLD patients (8 women and 11 men) and 18 control, non-NAFLD subjects (11 women and 7 men). The two subgroups were age- and sex-matched. All the recruited subjects underwent the following, after signing informed consent: (i) liver US, (ii) clinical and anthropometric evaluations, and (iii) blood draw for biochemical assessments and PBMC isolation. The diagnosis of NAFLD was based on liver US, performed by an experienced gastroenterologist. Body mass index (BMI) was calculated as participants’ weight in kilograms divided by the square of height in meters. Fatty liver index was calculated as described [9].

### 2.2. Measurement of Laboratory Data

Blood was collected in fasting conditions. Lithium-heparin tubes were used to measure plasma glucose, total cholesterol, LDL cholesterol, HDL cholesterol, triglycerides, high-sensitivity C-reactive protein (hs-CRP), ALT, AST, gamma-glutamyl transferase (GGT), and total bilirubin. Fasting insulin and haptoglobin were detected in the serum. Commercially available assays were used according to the manufacturer’s instructions (Roche diagnostics, Monza, Italy). All exams were performed on instruments calibrated against an appropriate proprietary reference standard material and verified using the registered quality controls. The homeostasis model assessment (HOMA) index [18] was calculated as the product of the fasting plasma insulin level (µU/mL) and the fasting plasma glucose level (mmol/L) divided by 22.5.

### 2.3. Isolation of PBMCs

Venous blood was collected in the morning under fasting conditions into S-Monovette K3E, 2.6 mL, containing 1.6 mg EDTA/mL (Sarstedt AG & Co., Nϋmbrecht, Germany). PBMCs were isolated from whole blood samples within two hours using the Sepmate™ method (Stemcell Technologies, Vancouver, Canada), following the manufacturer’s instructions. The Lymphoprep™ 1.077 g/mL density gradient medium (Stemcell Technologies) was added at a volume of 4.5 mL to the Sepmate™ 15 tube by carefully pipetting it through the central hole of the Sepmate™ insert. Blood (1.5 mL) was diluted with an equal volume of phosphate-buffered saline (PBS) (Immunological Science, Bolney, Sussex) containing 2% fetal bovine serum (FBS) (Immunological Science, Bolney, Sussex) and slowly layered on top of the Lymphoprep™ gradient. The tube was then spun at 1200× *g* for 10 min with the break on. The plasma layer was aspirated off, and the PBMC layer was collected. PBMCs were washed twice with fresh PBS/2%FBS medium by centrifuging at 300× *g* for 8 min. Cells were counted and viability checked with trypan blue staining using the TC20 automated cell counter (Bio-Rad Laboratories, Hercules, CA, USA) and then immediately used for the Extracellular Flux Analysis. Residual PBMC pellets were stored at −80 °C for subsequent mitochondrial DNA (mtDNA) quantification.

### 2.4. Extracellular Flux Analysis of PBMCs

Mitochondrial respirometry analysis of the PBMCs was determined using the Seahorse XFe24 Extracellular Flux Analyzer (Agilent, Santa Clara, CA, USA). This instrument provides a 24-well, fully automated format. Seahorse XFe24 cell culture microplates are combined with XFe24 sensor cartridges, containing probes that are lowered during the assay, forming a transient micro-chamber within each well, allowing changes in oxygen level and proton concentration to be detected. Cartridges also contain four ports per well, enabling the injection of modulators during the assay to measure key respiratory parameters. 

Isolated PBMCs were resuspended in Seahorse XF Base Medium (Agilent) by adding 5.5 mM glucose, 2 mM L-glutamine, and 1 mM sodium pyruvate and adjusting the pH at 7.4. Cells were seeded in quadruplicate at pre-determined cell densities (600,000 cells/well) in 200 μL/well on Seahorse XFe24 V7 PS Cell Culture Microplate (Agilent) pre-coated with Corning^®^ Cell-Tak™ cell and tissue adhesive (Corning, Discovery Labware, Bedford, MA). Microplates were centrifuged twice at 200× *g* for 1 min with the break off (with the microplate position rotated 180 degrees after the first centrifugation) to aid uniform cell attachment [19]. The medium volume was brought up to 525 μL in each well. Finally, microplates were incubated at 37 °C in a non-CO_2_ incubator for 1 h before running the assay.

We applied the Seahorse XF Cell Mito Stress test (Agilent) to measure cell oxygen consumption rate (OCR). Following the manufacturer’s instructions, the protocol was set to add mitochondrial modulators (75 μL/injection port) sequentially. Measurements in the absence of inhibitors define basal respiration. The ATP synthase inhibitor oligomycin (2 μM final concentration) was injected to retrieve proton leak values. The mitochondrial uncoupler carbonyl cyanide-4-(trifluoromethoxy)phenylhydrazone (FCCP, 1 μM) was then injected to measure maximal respiratory capacity. Finally, a mixture of the respiratory complex I inhibitor rotenone and the complex III inhibitor antimycin A (both at 0.5 μM) was injected to inhibit mitochondrial respiration completely. Data were retrieved and automatically analyzed using the Wave software (Agilent) after normalization for DNA content with the CyQUANT Cell Proliferation Assay Kit (Thermo Fisher Scientific, Waltham, MA, USA), according to the manufacturer’s protocol. All mitochondrial respiration values were measured after correcting for non-mitochondrial respiration. ATP-linked respiration was calculated as basal respiration minus the proton leak values. Spare respiratory capacity was calculated as maximal respiration minus the basal respiration values [16].

### 2.5. Mitochondrial DNA Quantification

Total DNA was extracted from PBMCs using Tissue Genomic DNA Extraction Kit (Fisher Molecular Biology, Rome, Italy), according to the manufacturer’s instructions. DNA concentration was quantified using the mySpec microvolume spectrophotometer (VWR International., Milan, Italy). mtDNA content was assessed by quantification of the human gene of the mitochondrially encoded NADH dehydrogenase 1 (ND1) relative to the nuclear gene β-2 microglobulin (β2-m). A real-time quantitative PCR (qPCR) assay was performed with the iTaq Universal SYBR Green Supermix (Bio-Rad) on the ViiA7 Real-Time PCR system (Applied Biosystems). The 20 μL volume reaction mixture contained 20 ng DNA, 10 μL iTaq Universal SYBR Green Supermix (Bio-Rad), and 0.5 µM primers (forward and reverse). All the sample reactions were performed in triplicate. The following protocol was performed: initial “hot start” activation step at 95 °C for 5 min, followed by 40 cycles at 95 °C for 15 s and at 60 °C for 1 min using the ViiA7 instrument (Applied Biosystems). We used the following primer pairs (5′-3′): ND1 forward (GTCAACCTCGCTTCCCCACCCT) and reverse (TCCTGCGAATAGGCTTCCGGCT) for mtDNA and β2-m forward (CGACGGGAGGGTCGGGACAA) and reverse (GCCCCGCGAAAGAGCGGAAG) for nuclear DNA [20]. The ratio of mtDNA to nuclear DNA was quantified as described [21]. We obtained the average of triplicate threshold cycle (Ct) values of the mtDNA and nDNA from each case. In the case of a standard deviation (SD) more than 0.2, the outlier value was suppressed for the analysis. Then, we calculated the ΔCt of average mtDNA and nDNA Ct values (ΔCt  = mtDNA Ct −  nDNA Ct). Mitochondrial DNA copy number was then calculated as 2 × 2^ΔCt^). Variables were log transformed to reduce skewness [21].

### 2.6. Measurement of Serum Cytokine IL-6 and TNF-α

We measured human serum TNF-α and IL-6 using two ELISA kits, Human TNF-alpha Quantikine HS ELISA (HSTA00E, R&D Systems, Minneapolis, MN, USA) and Human IL-6 Quantikine HS ELISA Kit (HS600C, R&D Systems, Minneapolis, MN, USA), respectively, according to the manufacturer’s instructions.

### 2.7. Statistical Analysis

Descriptive statistics were computed on variables of different natures. For quantitative variables, the number of missing values (N-Miss); mean and standard deviation (SD); median; first quartile (Q1) and third quartile (Q3); and range (minimum–maximum) were computed. In the case of categorical variables, frequencies (absolute and percentage values) were computed. Data were also stratified for the diagnosis (NAFLD/non-NAFLD) to identify any significant differences (*p*-value < 0.05) between the two subpopulations obtained. In doing this, the Wilcoxon Rank Sum Test (for quantitative variable) and the Fisher exact test (for qualitative variables) were applied. 

Mitochondrial respirometry variables were z-scored before conducting descriptive statistics. This transformation standardizes the data across a wide range of experiments and has been applied as a reliable procedure for respirometry analysis in many studies [21,22,23].

Spearman correlations ($\rho_{s}$) between couples of quantitative variables were visualized by means of a correlation plot since this is the best way to show relationships between the data. Blue and red circles correspond to positive and negative correlations, respectively [24,25,26,27]. The circle diameters and the color intensities are proportional to the magnitude of Spearman indexes; moreover, the black crosses on them identify correlations not significantly different from zero (in other terms, *p*-values > 0.05).

To identify which of the variables collected on the sample are determinant on the diagnosis of NAFLD, a data mining technique [28] was used. A Random Forest [29] with *k* = 10,000 trees was grown; the diagnosis (NAFLD/non-NAFLD) was the outcome (*Y*), and the clinical, biochemical, and mitochondrial variables were the covariates (*X*).

**Figure 1:**
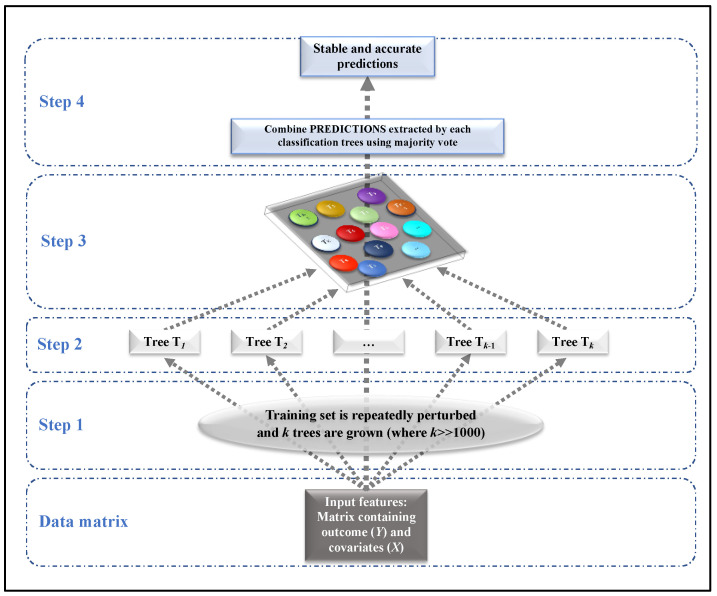
Steps involved in Random Forest (case of classification).

Random Forest is a supervised machine learning algorithm, widely used in classification problems in medicine [27,30,31,32], able to deal with continuous or categorical variables. This algorithm was introduced for solving the problem of instability of a single tree (small changes in the data induce big changes in the results), providing accurate predictions. It builds many classification trees on different samples of data and takes their majority vote for classification. Figure 1 summarizes how Random Forest works in 4 steps:

Step 1: The data matrix is repeatedly perturbed, obtaining k different subsets of observations (usually k = 10,000).
Step 2: For each perturbed subset obtained at Step 1, a single classification tree is grown.
Step 3: Each classification tree provides predictions (classes) in correspondence to each subject.
Step 4: Final prediction, which is stable and accurate, is obtained based on majority voting.

This model was only estimated with descriptive purpose and not for making predictions (accuracy metrics were not considered). An interesting tool of the Random Forest is the relative variable importance Measure (VIM_rel_) [33,34]. It identifies which are the most important variables in classifying patients as NAFLD/non-NAFLD. VIM is the mean decrease of accuracy over all out-of-bag (OOB) cross validated predictions, when a given variable is permuted after training but before prediction. VIM_rel_ standardizes the values obtained in correspondence of each covariate for simplifying the interpretation of the results; it is defined as the percent improvement with respect to the most important predictor, which has an importance of 100% (remaining variables have lower scores). Usually, VIM_rel_ is visualized by means of a lollipop plot, where in *x*-axis are reported the VIM_rel_ and in *y*-axis the corresponding covariates. This graph is basically a barplot where the bar is a substitute with a line and a dot; it displays the variables in order of their effect on model predictions (from least, VIM_rel_ ≅ 0%, to the most, VIM_rel_ = 100%, important variable).

Using the VIM_rel_, there is a simple way to make variable selection: all variables whose VIM_rel_ is ≥ than the median (Vim_rel_) are automatically selected.

All the analyses related to descriptive statistics or machine learning methods were performed with R, version 4.2.0 (R Foundation, Vienna, Austria). Statistical significance was set at *p*-value < 0.05. 

## 3. Results

### 3.1. Descriptive Statistics

#### 3.1.1. Clinical, Anthropometric, and Biochemical Characteristics of the Subjects

The baseline characteristics of the study cohort, with NAFLD and non-NAFLD control conditions diagnosed through abdominal US, are reported in Table 1. As expected, the NAFLD group had a statistically significant, increased waist circumference and BMI (all with *p*-value < 0.001) in comparison to the control group. Furthermore, NAFLD patients showed increased levels of fasting glucose (*p*-value = 0.014) and insulin (*p*-value < 0.001) compared with controls. Accordingly, the HOMA-index was higher in NAFLD patients, demonstrating a state of insulin-resistance. Total cholesterol (*p*-value = 0.027), triglycerides (*p*-value = 0.006), and LDL-cholesterol (*p*-value = 0.008) were augmented in NAFLD patients, while HDL-cholesterol values (*p*-value = 0.004) were reduced in NAFLD patients compared to controls. In accordance with the vast literature, no significant differences were observed between the two groups when comparing ALT, AST, GGT, total bilirubin, and haptoglobin (all *p*-values > 0.05). 

Interestingly and in line with recent works [7], inflammatory markers, such as hsCRP (*p*-value = 0.007), TNF-α (*p*-value = 0.025), and IL-6 (*p*-value = 0.006), were significantly higher in NAFLD patients compared to controls.

Finally, we found that the fatty liver index was strongly and significantly higher in NAFLD patients than in non-NAFLD ones (*p*-value < 0.001), confirming the diagnosis based on abdominal US.

#### 3.1.2. Mitochondrial DNA Content and Function in PBMCs from the Study Cohort

**Figure 2:**
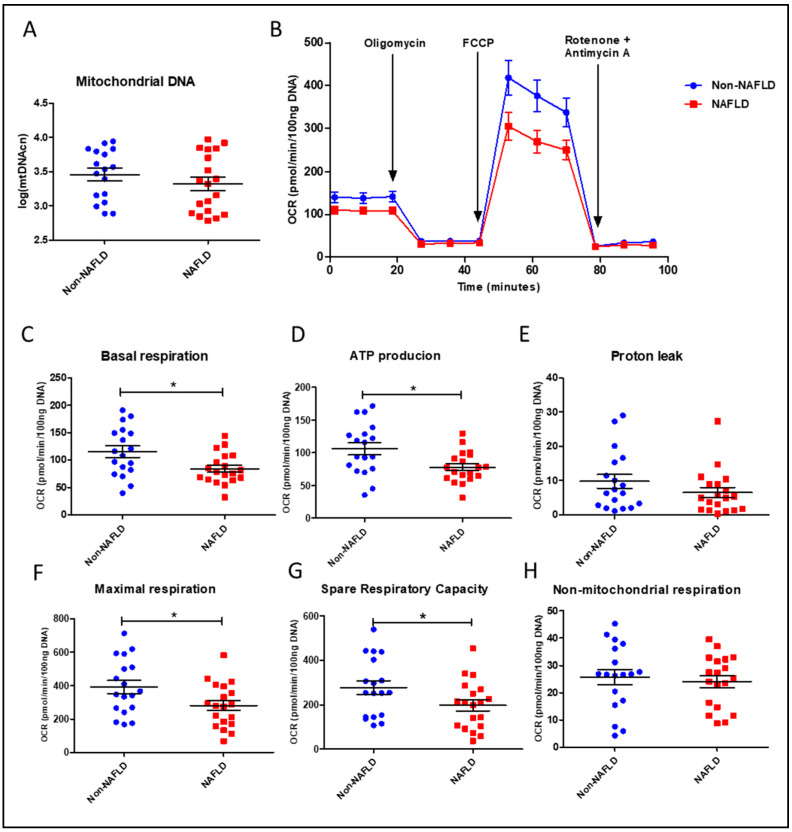
Mitochondrial content and function in PBMCs from NAFLD patients and non-NAFLD controls. (**A**) Quantification of mitochondrial DNA copy number (mtDNAcn) with log-transformed values. (**B**) Time-course representation of oxygen consumption rate (OCR) measured in live PBMCs during the Mito Stress Test. Three measurements at different times were performed for each condition by the Seahorse XFe24 analyzer. OCR values were normalized to DNA content. (**C**–**H**) Scatter dot plots of OCR values from individual subjects, normalized to DNA content, for each respiratory parameter. Data represent mean ± SEM. Statistical analysis has been performed with Wilcoxon Rank Sum Test. In scatter dot plots, * corresponds to *p*-value < 0.05. Statistical analysis was performed using GraphPad Prism 8.0.1 (San Diego, CA, USA).

The determination of PBMC mtDNA copy number showed no significant difference between the non-NAFLD and NAFLD cohorts (Table 2 and Figure 2A). We investigated mitochondrial function in live PBMC from the two cohorts by performing extracellular flux analysis for cellular bioenergetics. As described in Table 2, NAFLD subjects had significantly lower mitochondrial respiratory parameters in blood cells compared to the non-NAFLD cohort. Of note, NAFLD patients exhibited lower basal respiration (*p*-value = 0.023), ATP-production associated respiration (*p*-value = 0.021), maximal respiration (*p*-value = 0.045), and spare respiratory capacity (*p*-value = 0.045) compared to the non-NAFLD cohort. No significant difference was found in non-mitochondrial respiration and proton leak between the two cohorts (Table 2). The two latter parameters are referred to as (*i*) oxygen consumption due to cellular processes involving the activity of various non-mitochondrial enzymes; and (*ii*) residual oxygen consumption often referred to as “inefficient respiration” [16] whose nature is not fully understood and whose interpretation is not feasible without applying additional techniques [35].

Mitochondrial bioenergetics parameter data are also visualized in Figure 2B–H. Figure 2B describes the real-time pattern of PBMC oxygen consumption measured during the Mito Stress Test after adding the specific mitochondrial inhibitors. Figure 2 C–H shows scatter dot plots of individual data points for each respirometry parameter automatically retrieved by the Wave software. Our results indirectly confirm that mitochondrial derangements are involved in NAFLD, as investigated by various research groups in the liver [12,13]. Moreover, the reduction of basal, ATP-production associated, maximal, and “spare” or reserve respiration in live circulating PBMCs from NAFLD patients suggests that key respiratory parameters, measured in blood cells, could mirror the course of this disease with systemic involvement and could be adopted as novel disease biomarkers. Our next step was to investigate the relationships among these mitochondrial bioenergetic parameters and the other clinical, anthropometric, and biochemical characteristics in the whole study cohort. Due to their limited and/or controversial use in mitochondrial function studies [16,35], non-mitochondrial respiration and proton leak-linked respiration were excluded from further analyses.

#### 3.1.3. Correlation

**Figure 3:**
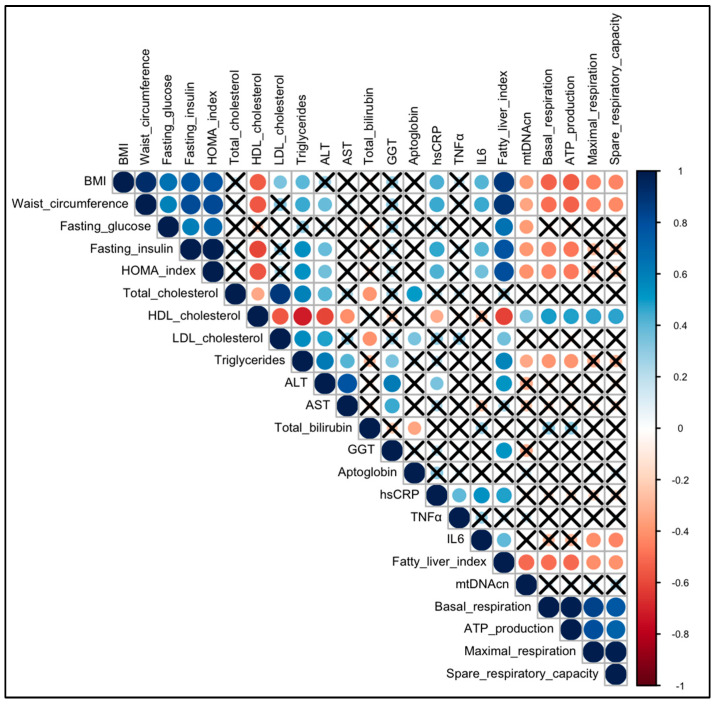
Correlation plot for quantitative variables collected in this study. This correlation plot visualizes all the Spearman correlations ($\rho_{s}$) between couples of quantitative variables collected in this study. Blue and red circles correspond to positive (0 ${\leq \rho }_{s}\leq$ 1) and negative correlations (${-1\leq \rho }_{s}<$ 0), respectively. The diameter and the color intensity of the circle is proportional to the magnitude of the Spearman index, and the black cross identifies it as not a significant correlation (correlation test *p*-values > 0.05). ALT, alanine aminotransferase; AST, aspartate aminotransferase; BMI, Body Mass Index; GGT, gamma-glutamyl transferase; HOMA index, homeostasis model assessment index; hsCRP, high-sensitivity C-reactive protein; IL6, interleukin-6; mtDNAcn, mitochondrial DNA copy number; TNFα, tumor necrosis factor-α.

An overview of the pairwise Spearman correlations ($\rho_{s}$) between quantitative variables involved in the study (considering the entire sample) is visualized in Figure 3 with a correlation plot were the magnitude and direction (${0\leq \rho }_{s}\leq 1\to positive;{-1\leq \rho }_{s}<0\to negative$) of the correlations are reflected by the size (larger is stronger) and color (blue is positive and red is negative) of the circles, respectively. There are significant (*p*-values < 0.05) negative correlations between BMI and the following: mtDNAcn ($\rho_{s}$ = −0.37; *p*-value = 0.024), basal respiration ($\rho_{s}$ = −0.52; *p*-value = 0.001), ATP production ($\rho_{s}$ = −0.54; *p*-value = 0.001), maximal respiration ($\rho_{s}$ = −0.44; *p*-value = 0.006), spare respiratory capacity ($\rho_{s}$ = −0.44; *p*-value = 0.007). Significant negative correlations were identified between waist circumference and the following: mtDNAcn ($\rho_{s}$ = −0.34; *p*-value = 0.048), basal respiration ($\rho_{s}$ = −0.49; *p*-value = 0.003), ATP production ($\rho_{s}$ = −0.52; *p*-value = 0.001), maximal respiration ($\rho_{s}$ = −0.43; *p*-value = 0.009), spare respiratory capacity ($\rho_{s}$ = −0.43; *p*-value = 0.011). Significant negative correlations were found between the HOMA index and the following: mtDNAcn ($\rho_{s}$ = −0.41; *p*-value = 0.014), basal respiration ($\rho_{s}$ = −0.43; *p*-value = 0.008), ATP production ($\rho_{s}$ = −0.47; *p*-value = 0.004). There are significant negative correlations between fasting insulin and the following: mtDNAcn ($\rho_{s}$ = −0.39; *p*-value = 0.020), basal respiration ($\rho_{s}$ = −0.44; *p*-value = 0.006), ATP production ($\rho_{s}$ = −0.48; *p*-value = 0.003). Again, there are significant negative correlations between triglycerides and the following: mtDNAcn ($\rho_{s}$ = −0.33; *p*-value = 0.047), basal respiration ($\rho_{s}$ = −0.40; *p*-value = 0.017), ATP production ($\rho_{s}$ = −0.31; *p*-value = 0.015). It is interesting to note that there are significant positive correlations between HDL cholesterol and the following: mtDNAcn ($\rho_{s}$ = +0.35; *p*-value = 0.039), basal respiration ($\rho_{s}$ = +0.49; *p*-value = 0.002), ATP production ($\rho_{s}$ = +0.49; *p*-value = 0.002), maximal respiration ($\rho_{s}$ = +0.48; *p*-value = 0.003), spare respiratory capacity ($\rho_{s}$ = +0.48; *p*-value = 0.003).

No significant correlations were found with other lipid markers, such as total cholesterol, LDL cholesterol, and with classical liver markers, such as AST, ALT, and GGT. Moreover, the unique mitochondrial marker that is negatively correlated with fasting glucose is mtDNAcn ($\rho_{s}$ = −0.38; *p*-value = 0.023).

Except for IL-6, which is inversely correlated with maximal respiration ($\rho_{s}$ = −0.42; *p*-value = 0.010) and spare respiratory capacity ($\rho_{s}$ = −0.42; *p*-value = 0.008), no other significant correlations were noted for the remaining inflammatory markers in the study (TNF-α and hsCRP). Finally, the fatty liver index is inversely correlated with mitochondrial markers with a *p*-value < 0.05 for all the mitochondrial markers analyzed.

#### 3.1.4. Random Forest and VIMrel

With the purpose of identifying which of the variables collected in this study are determinant on the diagnosis of NAFLD, Random Forest was used where the outcome (*Y*) was the diagnosis (NAFLD/non-NAFLD) and the covariates (*X*) were the clinical, biochemical, and mitochondrial variables. In detail:

Diagnosis = fasting glucose + total cholesterol + HDL cholesterol + LDL cholesterol + triglycerides + ALT + AST + total bilirubin + GGT + haptoglobin + hs-CRP + TNF-α + IL-6 + mtDNAcn + basal respiration + ATP production + maximal respiration + spare respiratory capacity.

Clinical variables and insulin were excluded by this analysis since they are clearly associated with the diagnosis, and they would mask the remaining variables of the model.

**Figure 4:**
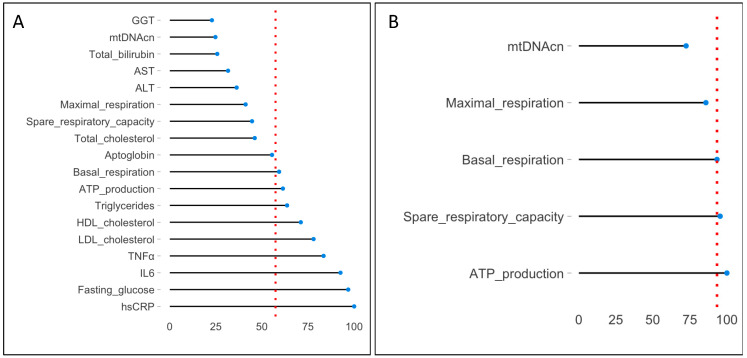
VIM_rel_ extracted from two Random Forest models. The VIM_rel_ is an interesting tool provided by Random Forest which assigns a percentage (from a minimum of 0% to a maximum of 100%) to each covariate in the model. The lollipop graph reports these percentages on the *x*-axis and the predictors (variables) on the *y*-axis. Variables are reordered in ascending importance in predicting the diagnosis (the least important at the top of the graph, the most important, with a VIM_rel_ = 100%, at the bottom of the graph). The dashed red line is the median (VIM_rel_), which represents the cut-off point for making variable selection. (**A**) shows the VIM_rel_ of the first model involving biochemical and mitochondrial variables. The cut-off point for variable selection is 57.36%, and covariates that exceeded (or are equal to) this threshold are hsCRP, fasting glucose, IL-6, TNF-α, LDL cholesterol, HDL cholesterol, triglycerides, ATP production, and basal respiration. (**B**) shows the VIM_rel_ of the second model involving only mitochondrial variables. Here, the cut-off point for variable selection is 93.32%, and covariates that exceeded (or are equal to) this threshold are ATP production, spare respiratory capacity, and basal respiration. Abbreviations are like those in the legend to Figure 3.

The VIMrel, which assigns a percentage (from a minimum of 0% to a maximum of 100%) to each covariate in the model, was extracted from the Random Forest. These values were visualized by a lollipop graph (Figure 4A). It reports in ascending order all the variables used in the model (at the top of the graph is the least important variable, GGT with a VIMrel = 22.84%, while at the bottom is the most important variable, hsCRP with a VIMrel = 100.00%). The dashed red line is the median value of the VIMrel (equals to 57.35%); all the variables with VIMrel ≥ 57.35% are determinant in the prediction of the diagnosis. In detail: hsCRP (VIMrel = 100%), fasting glucose (VIMrel = 96.78%), IL-6 (VIMrel = 92.58%), -TNF-α (VIMrel = 83.41%), LDL cholesterol (VIMrel = 77.98%), HDL cholesterol (VIMrel = 71.06%), triglycerides (VIMrel = 63.62%), ATP production (VIMrel = 61.35%), and basal respiration (VIMrel = 59.27%). This means that clinicians could focus their attention on a limited number of variables during the diagnosis. It is interesting to note that two of them are mitochondrial variables (ATP production and basal respiration with a VIMrel of 61.35% and 59.27%, respectively), which, together with biochemical variables, improve the decision-making process that leads to the classification of a patient as NAFLD/non-NAFLD.

To study the impact of mitochondrial variables on the diagnosis, a second Random Forest was estimated, excluding the effect of biochemical variables:

Diagnosis = mtDNAcn + basal respiration + ATP production + maximal respiration + spare respiratory capacity 

Figure 4B reports the VIMrel extracted from this second model. Here, the cut-off point for variable selection is 93.32%. The covariates with VIMrel that exceed (or are equal to) this threshold are ATP production (VIMrel = 100%), spare respiratory capacity (VIMrel = 95.44%), and basal respiration (VIMrel = 93.32%). This result supports the hypothesis that mitochondrial impairment is important in NAFLD, especially in terms of a reduced ability to cope with cellular energy demand.

## 4. Discussion

The measurement of mitochondrial bioenergetic parameters in circulating blood cells is emerging, not only as a minimally invasive method in clinical research (particularly in large clinical trials with repeated assessments at multiple time points), but also as a promising method for diagnostic developments and therapeutic monitoring in personalized medicine [17]. We have applied this approach to NAFLD, a highly prevalent chronic condition currently devoid of noninvasive, well-defined diagnostic tools. By applying extracellular flux analysis-based respirometry in live circulating PBMCs, we found significant impairment in key mitochondrial respiratory parameters in NAFLD adult patients compared to age- and sex-matched controls. In particular, after correcting for non-mitochondrial respiration, basal respiration (representing the contribution of mitochondrial respiration in response to the steady-state energy demand of the cell) and ATP-linked respiration (the critical component of basal respiration to drive mitochondrial ATP synthesis) were significantly reduced in PBMCs from NAFLD cases. Most interestingly, maximal respiration and spare respiratory capacity (the difference between maximal and basal respiration, also called respiratory reserve capacity, indicating the ability of the cell to meet increased energy demand) were significantly decreased in NAFLD-derived PBMCs. 

Our data are in line with recently published ones, also describing a progressive impairment of PBMC maximal respiration correlating to the extent of fibrosis in NAFLD patients [36]. Notably, NAFLD progression from simple steatosis to NASH, with substantial fibrosis, is mediated by inflammatory cytokines, including IL-1β, IL-6, and TNFα, overproduced by resident liver macrophages, i.e., the inflammatory-polarized M1-Kupffer cells [37]. Recent studies also reported that PBMCs of NAFLD patients are more sensitive to gut-derived pathogen-associated molecular patterns (PAMPs) and release higher amounts of proinflammatory cytokines (IL-1β and IL-6) upon PAMP exposure than PBMCs derived from controls [38,39]. This response was concentration-dependent and correlated with the extent of hepatic injury [39]. Of interest, we observed that IL-6 plasma levels (which were significantly increased in NAFLD patients and positively correlated with BMI, waist circumference, fasting insulin, HOMA index, and hs-CRP) showed a negative correlation with PBMC maximal respiration and spare respiratory capacity in our cohort. Our results suggest that IL-6, related to the degree of hepatic inflammation and stage of fibrosis [14], could be one of the main drivers for impairing mitochondrial respiration in the liver and blood cells of NAFLD patients.

We found that PBMC absolute mtDNAcn, an index of mitochondrial mass, correlated negatively with unhealthy indicators (e.g., obesity-related anthropometric measurements, triglyceride levels, and indexes of insulin resistance such as fasting glucose and insulin levels and HOMA index) in the whole cohort. These correlations emphasize the importance of introducing a simple and inexpensive parameter, such as qPCR-based quantification of mitochondrial mass, into a comprehensive panel of biomarkers for the diagnosis of NAFLD. Although this parameter did not emerge as significantly different between healthy and affected subjects, it appears to have a trend comparable to current disease biomarkers within the total population. This finding could be explained by the fact that the division into NAFLD patients and controls dichotomizes what is instead a disease progression characterized by different stages, masking possible nonlinear variation in the parameters involved. 

The mitochondrial respiratory pattern of correlations with other biochemical and clinical markers suggests their involvement in glucose and lipid homeostasis systems. Basal and ATP-linked respiration showed a negative correlation with fasting insulin levels and the HOMA index, reinforcing the long-known role of mitochondrial dysfunction in the pathophysiology of insulin resistance and associated metabolic complications in liver and other organs [40,41].

Levels of circulating lipids affect mitochondrial function [42], including PBMC respiratory capacity. We observed that HDL levels positively correlated with mtDNAcn and with all the mitochondrial respiratory parameters (basal respiration, ATP production, maximal respiration, and spare respiratory capacity). On the contrary, triglyceride levels negatively correlated with mtDNAcn and basal and ATP-linked respiration. In our samples, no significant correlations were found between LDL and mitochondrial respiration. This observation deserves further attention. A recent study demonstrated that NAFLD features lower serum lipid species containing polyunsaturated fatty acids, the most affected lipid fractions being HDL [43]. We did not explore changes in the gut microbiome, which is mainly modulated by diet, deeply affecting metabolic health [44]. Of interest, the gut microbiome is a major determinant of blood lipids, specifically triglycerides and HDL cholesterol [45], and affects mitochondrial function [46]. A large consensus of experts has recently proposed to rename NAFLD as metabolic (dysfunction)-associated fatty liver disease (MAFLD) [4], mainly based on pathophysiological evidence and recognizing that it represents the hepatic manifestation of a systemic metabolic disorder. Though not yet universally accepted, this new definition also highlights the contribution of cardiometabolic risk factors to the development and progression of liver disease [1], and our findings corroborate this view.

The main finding in our study is the identification of novel potential biomarkers of increased risk of NAFLD. This is of paramount importance since today, there are no accurate, noninvasive methods to diagnose and monitor disease progression. In various scenarios, the measurement of circulating markers facilitates and/or completes an early diagnosis and may give insight to illness evolution, with important advantages, such as cost, availability, or waiting time, when compared to current diagnostic approaches [47,48,49]. Though not yet used diffusely in clinical laboratory settings, respirometry analysis is rapidly expanding, showing a high potential for its application in routine diagnostics [16].

Our study has limitations due to its cross-sectional design and the relatively small size of participants. Further, larger studies, with longitudinal follow-up, would strengthen our results about the relationships between blood cell mitochondrial respiratory parameters and other circulating biomarkers to aid in NAFLD/MAFLD diagnosis. Therapeutic approaches aimed at preserving liver mitochondrial function are currently being explored [50,51]. If confirmed and extended, measurements of PBMC mitochondrial (dys)function could be included in panels of NAFLD diagnostic biomarkers to monitor disease progression and the response to current and novel therapies, including mitochondrial-targeted ones.

## Figures and Tables

**Table 1:** Descriptive statistics on clinical variables and analytes of the study cohorts.

Variables	NAFLD	Non-NAFLD	Total	*p* Value
	(N = 19)	(N = 18)	(N = 37)	
**Sex**				0.330 ^(a)^
Female	8 (42.1%)	11 (61.1%)	19 (51.4%)	
Male	11 (57.9%)	7 (38.9%)	18 (48.6%)	
Age (years)				0.068 ^(b)^
Mean (SD)	51.84 (4.15)	48.44 (5.74)	50.19 (5.21)	
Median (Q1, Q3)	52.00 (50.00, 54.50)	48.00 (43.25, 52.75)	51.00 (47.00, 54.00)	
Range	42.00–59.00	40.00–59.00	40.00–59.00	
BMI (Kg/m^2^)				***<0.001*** ^(b)^
Mean (SD)	32.62 (6.93)	23.49 (3.64)	28.18 (7.18)	
Median (Q1, Q3)	31.30 (27.85, 34.55)	23.40 (20.50, 25.77)	27.00 (23.50, 31.50)	
Range	25.20–55.00	17.60–31.70	17.60–55.00	
Waist Circumference (cm)				***<0.001 *** ^(b)^
N-Miss	0	2	2	
Mean (SD)	107.34 (17.71)	86.97 (9.42)	98.03 (17.64)	
Median (Q1, Q3)	105.00 (96.50, 112.00)	87.50 (81.00, 94.62)	96.00 (88.00, 106.25)	
Range	86.00–169.00	70.00–99.00	70.00–169.00	
Fasting glucose (mg/dL)				***0.014 *** ^(b)^
Mean (SD)	99.37 (22.30)	86.39 (11.26)	93.05 (18.76)	
Median (Q1, Q3)	91.00 (86.00, 105.00)	81.50 (77.75, 95.25)	88.00 (81.00, 98.00)	
Range	81.00–169.00	74.00–115.00	74.00–169.00	
Fasting insulin (µUI/mL)				***<0.001 *** ^(b)^
Mean (SD)	14.21 (9.76)	4.44 (3.38)	9.46 (8.81)	
Median (Q1, Q3)	12.00 (8.50, 18.00)	4.00 (2.00, 5.00)	8.00 (4.00, 13.00)	
Range	2.00–42.00	1.00–14.00	1.00–42.00	
HOMA index				***<0.001 *** ^(b)^
Mean (SD)	3.60 (2.78)	1.02 (0.95)	2.34 (2.45)	
Median (Q1, Q3)	2.86 (1.72, 4.69)	0.81 (0.40, 1.16)	1.60 (0.79, 3.11)	
Range	0.42–11.27	0.18–3.98	0.18–11.27	
Total cholesterol (mg/dL)				***0.027 *** ^(b)^
Mean (SD)	209.37 (28.51)	185.94 (31.09)	197.97 (31.68)	
Median (Q1, Q3)	212.00 (189.50, 230.00)	185.00 (167.75, 200.25)	195.00 (176.00, 218.00)	
Range	165.00–262.00	117.00–259.00	117.00–262.00	
HDL cholesterol (mg/dL)				***0.004 *** ^(b)^
Mean (SD)	51.36 (13.93)	67.49 (16.96)	59.21 (17.31)	
Median (Q1, Q3)	47.60 (39.65, 62.80)	69.50 (53.32, 75.33)	55.60 (46.10, 71.80)	
Range	33.20–77.60	43.30–112.80	33.20–112.80	
LDL cholesterol (mg/dL)				***0.008 *** ^(b)^
Mean (SD)	131.53 (29.39)	102.72 (31.99)	117.51 (33.59)	
Median (Q1, Q3)	127.00 (118.00, 154.50)	95.50 (86.25, 118.75)	119.00 (94.00, 142.00)	
Range	72.00–194.00	37.00–166.00	37.00–194.00	
Triglycerides (mg/dL)				***0.006 *** ^(b)^
Mean (SD)	132.37 (73.47)	78.78 (45.27)	106.30 (66.37)	
Median (Q1, Q3)	126.00 (71.00, 155.00)	64.00 (49.75, 91.75)	77.00 (60.00, 131.00)	
Range	57.00–301.00	32.00–219.00	32.00–301.00	
ALT (U/L)				0.163 ^(b)^
N-Miss	0	1	1	
Mean (SD)	33.89 (14.49)	31.12 (23.40)	32.58 (18.98)	
Median (Q1, Q3)	32.00 (22.50, 40.50)	23.00 (18.00, 32.00)	30.50 (19.00, 35.50)	
Range	13.00–64.00	15.00–108.00	13.00–108.00	
AST (U/L)				0.867 ^(b)^
Mean (SD)	16.79 (6.24)	18.28 (10.81)	17.51 (8.67)	
Median (Q1, Q3)	16.00 (11.50, 22.00)	14.50 (10.25, 22.75)	15.00 (11.00, 22.00)	
Range	8.00–27.00	6.00–43.00	6.00–43.00	
Total bilirubin (mg/dL)				0.648 ^(b)^
Mean (SD)	1.36 (3.43)	0.66 (0.29)	1.02 (2.46)	
Median (Q1, Q3)	0.55 (0.48, 0.66)	0.56 (0.53, 0.69)	0.56 (0.50, 0.66)	
Range	0.29–15.50	0.35–1.42	0.29–15.50	
GGT (U/L)				0.564 ^(b)^
Mean (SD)	33.58 (40.86)	27.85 (34.71)	30.79 (37.58)	
Median (Q1, Q3)	22.70 (11.60, 35.50)	16.40 (12.67, 21.55)	17.10 (12.40, 32.70)	
Range	0.50–178.60	5.30–152.40	0.50–178.60	
Haptoglobin (mg/dL)				0.098 ^(b)^
Mean (SD)	133.71 (57.92)	106.71 (48.92)	120.58 (54.73)	
Median (Q1, Q3)	148.00 (87.30, 170.00)	119.00 (68.10, 134.75)	121.00 (79.40, 165.00)	
Range	21.00–224.00	21.00–202.00	21.00–224.00	
hsCRP (mg/L)				***0.007 *** ^(b)^
Mean (SD)	3.26 (3.36)	1.32 (1.49)	2.31 (2.77)	
Median (Q1, Q3)	1.43 (1.02, 4.25)	0.61 (0.34, 1.70)	1.13 (0.56, 2.70)	
Range	0.33–10.00	0.08–4.96	0.08–10.00	
TNF-α (pg/mL)				***0.025 *** ^(b)^
N-Miss	0	1	1	
Mean (SD)	3.26 (1.64)	2.34 (1.32)	2.83 (1.55)	
Median (Q1, Q3)	3.02 (2.59, 3.38)	2.38 (1.47, 2.60)	2.60 (2.15, 3.37)	
Range	1.62–9.37	0.00–5.68	0.00–9.37	
IL-6 (pg/mL)				***0.006 *** ^(b)^
Mean (SD)	2.40 (2.11)	1.07 (0.73)	1.75 (1.72)	
Median (Q1, Q3)	1.55 (1.11, 2.70)	0.95 (0.61, 1.30)	1.19 (0.80, 1.58)	
Range	0.53–8.40	0.24–3.65	0.24–8.40	
Fatty Liver Index				***<0.001 *** ^(b)^
N-Miss	0	2	2	
Mean (SD)	67.95 (29.34)	20.13 (18.19)	46.09 (34.44)	
Median (Q1, Q3)	80.59 (55.52, 89.81)	14.53 (3.78, 33.95)	39.57 (14.53, 80.68)	
Range	3.35–98.61	1.32–60.69	1.32–98.61	

^(a)^ Fisher exact test ^(b)^ Wilcoxon Rank Sum Test. In bold and italics, *p*-values < 0.05. ALT, alanine aminotransferase; AST, aspartate aminotransferase; BMI, Body Mass Index; GGT, gamma-glutamyl transferase; HOMA, homeostasis model assessment; hs-CRP, high-sensitivity C-reactive protein; IL-6, interleukin-6; TNF- α, tumor necrosis factor-α.

**Table 2:** Descriptive statistics on mitochondrial parameters in the study cohorts.

Variables	NAFLD	Non-NAFLD	Total	*p* Value
	(N = 19)	(N = 18)	(N = 37)	
mtDNAcn				0.318 ^(a)^
N-Miss	0	1	1	
Mean (SD)	3.32 (0.43)	3.46 (0.37)	3.39 (0.40)	
Median (Q1, Q3)	3.32 (2.91, 3.76)	3.54 (3.15, 3.80)	3.39 (3.02, 3.80)	
Range	2.78–3.97	2.89–3.94	2.78–3.97	
Basal respiration				***0.023 *** ^(a)^
Mean (SD)	−0.37 (0.74)	0.45 (1.17)	0.03 (1.05)	
Median (Q1, Q3)	−0.52 (−0.84, 0.09)	0.37 (−0.39, 1.34)	−0.24 (−0.73, 0.63)	
Range	−1.74–1.22	−1.54–2.45	−1.74–2.45	
ATP production				***0.021 *** ^(a)^
Mean (SD)	−0.37 (0.71)	0.46 (1.17)	0.04 (1.04)	
Median (Q1, Q3)	−0.39 (−0.83, 0.10)	0.43 (−0.40, 1.12)	−0.11 (−0.63, 0.77)	
Range	−1.77–1.17	−1.64–2.41	−1.77–2.41	
Proton leak				0.191 ^(a)^
Mean (SD)	−0.19 (0.80)	0.22 (1.08)	0.01 (0.96)	
Median (Q1, Q3)	−0.39 (−0.81, 0.11)	−0.15 (−0.64, 0.79)	−0.27 (−0.75, 0.30)	
Range	−0.97–2.41	−0.87–2.62	−0.97–2.62	
Maximal respiration				***0.045 *** ^(a)^
Mean (SD)	−0.30 (0.81)	0.38 (1.01)	0.03 (0.96)	
Median (Q1, Q3)	−0.31 (−0.93, 0.28)	0.16 (−0.39, 1.08)	0.02 (−0.73, 0.57)	
Range	−1.60–1.52	−1.00–2.33	−1.6–2.33	
Spare respiratory capacity				***0.045*** ^(a)^
Mean (SD)	−0.26 (0.81)	0.33 (0.98)	0.02 (0.93)	
Median (Q1, Q3)	−0.18 (−0.94, 0.20)	0.16 (−0.63, 1.09)	−0.06 (−0.69, 0.55)	
Range	−1.46–1.63	−0.93–2.27	−1.46–2.27	
Non-mitochondrial respiration				0.715 ^(a)^
Mean (SD)	−0.06 (0.82)	0.08 (1.04)	0.01 (0.92)	
Median (Q1, Q3)	0.07 (−0.79, 0.60)	0.17 (−0.58, 0.86)	0.17 (−0.72, 0.64)	
Range	−1.37–1.26	−1.75–1.76	−1.75–1.76	

^(a)^ *p*-values of the Wilcoxon Rank Sum Test. In bold and italics, *p*-values < 0.05. mtDNAcn, mitochondrial DNA copy number.

## Data Availability

The data presented in this study are available on request from the corresponding author. The data are not publicly available due to privacy restrictions.
